# Dehydration, Wellness, and Training Demands of Professional Soccer Players during Preseason

**DOI:** 10.1155/2022/8054449

**Published:** 2022-11-07

**Authors:** César Leão, Francisco Tomás González-Fernández, Halil İbrahim Ceylan, Filipe Manuel Clemente, Hadi Nobari, Miguel Camões, José Maria Cancela Carral

**Affiliations:** ^1^University of Vigo, Faculty of Educational Sciences and Sports Sciences, 36005 Pontevedra, Spain; ^2^Escola Superior Desporto e Lazer, Instituto Politécnico de Viana do Castelo, Rua Escola Industrial e Comercial de Nun'Álvares, 4900-347 Viana do Castelo, Portugal; ^3^Research Center in Sports Performance, Recreation, Innovation and Technology (SPRINT), 4960-320 Melgaço, Portugal; ^4^Department of Physical Education and Sports, Faculty of Education and Sport Sciences, Campus of Melilla, University of Granada, 52006 Melilla, Spain; ^5^Physical Education and Sports Teaching Department, Kazim Karabekir Faculty of Education, Ataturk University, Erzurum, Turkey; ^6^Instituto de Telecomunicações, Delegação da Covilhã, Lisboa 1049-001, Portugal; ^7^Department of Exercise Physiology, Faculty of Educational Sciences and Psychology, University of Mohaghegh Ardabili, Ardabil 56199-11367, Iran; ^8^Department of Motor Performance, Faculty of Physical Education and Mountain Sports, Transilvania University of Braşov, 500068 Braşov, Romania; ^9^Faculty of Sport Sciences, University of Extremadura, 10003 Cáceres, Spain

## Abstract

**Purpose:**

Our study is aimed at analyzing the relationships between water loss and a professional soccer team's internal and external training load throughout the first three months of a season, covering all the preseason and the first two months of the competitive season.

**Methods:**

This study followed an observational analytic design. Twenty-seven athletes (age: 25.5 ± 4.1 years, height: 180.7 ± 8.2 cm, and body mass: 78.4 ± 8.7 kg) were included in the study, conducted over the first three months of the season. Players were weighed at the beginning and end of all training sessions to estimate fluid losses. They were asked to complete a wellness questionnaire and indicate the color of the first urine of the day upon their arrival at the practice session. Additionally, all sessions were monitored for locomotor demands.

**Results:**

We found a positive correlation between urine color and sprint distance (*r* = 0.46, *p* = 0.01) and a positive correlation between dehydration and rating of perceived exertion (*r* = 0.44, *p* = 0.015), whereas a negative correlation between dehydration and number of acceleration (*r* = −0.39, *p* = 0.034).

**Conclusions:**

Dehydration increased perceived physical exertion. Regularly monitoring training load and changes in body mass, as well as raising awareness about hydration, can contribute to cognitive and physical performance.

## 1. Introduction

Soccer is the most popular sport worldwide and is played by millions globally [1]. It can be characterized as a high-intensity intermittent sport, sporadic, and involving an infinity of physical actions that are reflected in the proficiency execution of various technical actions [2–4]. Over the years, many changes have occurred, with a significant impact on its physical demands [5, 6]. Today, the ability to perform not only low-intensity exercise but also (and especially) high-intensity exercise and explosiveness is a fundamental component of good performance by athletes [5–7]. Furthermore, at the highest level, the number of games has increased, meaning that the weekly congestion of competitive moments is also increasing [8, 9].

It is well known that good nutritional strategies are fundamental to the health and performance of athletes, as not only the food but also the fluids consumed will greatly influence athletes' performance and recovery [10, 11]. Training and playing can result in dehydration from ingesting fewer fluids lost through sweating [12]. This negative balance impacts not only the physical performance of athletes but also their mental and cognitive performance [13], particularly among soccer players [14]. Also, the level of fatigue that affects athletes seems to be related to some factors. Among them is the degree of hypohydration presented at the end of a game [15]. This dehydration-related decrease in performance may have several causes, such as increased body temperature or increased use of glycogen and the consequent decrease in muscle reserves [16, 17]. Because of that, a difference in the pattern of activity of the athletes has also been seen, as the distance covered during high-intensity movements is smaller when temperatures are higher, and dehydration is greater [18–20].

Considering the evolution of the physical demands of soccer, the science behind attempting to reduce the risk of injury for athletes and optimize their performance capacity has turned to controlling the training load. It is now the focus of attention of elite teams worldwide [21]. Training load can be represented in the form of internal load, which can be measured by the perception of effort, or in the form of external load, which can be presented as total distance, sprint distance, accelerations (ACCs), decelerations (DCC), and so on [22, 23].

Some authors have already related changes in body mass in an acute way as the representative of water losses with other external and internal load parameters [24]. However, to our knowledge, no such studies have been conducted over such a long period or while targeting games and training sessions. We believe that water loss that leads to dehydration can be reflected in poorer performance and analyzed recurring changes in internal and external load in athletes. Therefore, our study is aimed at analyzing the relationships between water loss and internal and external training load in a professional soccer team throughout the first three months of a season. We hypothesized that there would be a more significant training load for higher levels of dehydration during training and the game, a greater perception of effort associated with a decrease in the distance covered, and high-intensity efforts.

## 2. Materials and Methods

Body mass variations in training and elite soccer players' matches were analyzed during the season to evaluate fluid loss. Physical activity was also analyzed to estimate player load and correlate with the variable above.

### 2.1. Subjects

Twenty-seven male elite soccer players (age: 25.5 ± 4.1 years, height: 180.7 ± 8.2 cm, and body mass: 78.4 ± 8.7 kg) of the same Portuguese first league team participated in this study. Concerning the sample size, a minimum sample size of twenty-one participants was required for a power level of 81.7% (real power). We determined by an a priori power, *t*-test, correlation analysis with an *α* error of 0.05, power (1‐*β* err prob) 0.80, and medium-to-large effect size based on data from a previous study [12]. G∗Power software (University of Düsseldorf, Düsseldorf, Germany) was used to calculate sample power. These male elite soccer players trained five times a week (70 min per session) and played at least one match per week. The training sessions were based on technical and tactical content development, technical skill improvements, general improvements in physical condition, and recovery. Generally, training sessions comprised a warm-up, a prominent part, and a cooldown. All players were informed about the study protocol, assumptions, benefits, and risks, and their written consent forms were obtained before the study began. The ethics committee certified by the National Council of Health approved the study. The study was conducted according to the principles of the declaration of Helsinki.

### 2.2. Procedures

The measurements were done during the season's first three months, from July to September. This period covered all the preseason and the first two months of the competitive season. Regarding periodization, we divided this period into four macrocycles with the following characteristics: microcycle 1 (from week 1 to week 4), microcycle 2 (from week 5 to week 8), microcycle 3 (from week 9 to week 12), and microcycle 4 (from week 13 to week 16).

This corresponded to summertime when the mean air temperature was 21.70°C [28.3; 17.3]. Players were weighed in underwear using a 0.1 kg precise portable digital scale (model Seca 803, Medical measurement systems and scales, Hamburg, Germany) just before and immediately after each training session and each match (three minutes after every training session and match). Players were asked to do body mass after they emptied their bladder, and after the training session, they were asked to dry themselves before weighing. We get the total fluid loss by applying the difference between the final weight and the weight in the beginning [25]. The reason to use this difference is that even without taking into account the sweat rate of the athletes or the total amount of fluid drunk by the athlete, the difference remains that the total fluid loss in weight is primarily due to that reason [26].

Additionally, players were asked to fulfil a questionnaire when they arrived on the premises. In this questionnaire, they were asked to choose from a urine color chart the number corresponding to the color of their first urine in the morning [27]. This table has a scale of 1 to 8, where 1 to 3 correspond to dehydration, and from 4 to 8, we are progressively more dehydrated [28]. All players received instructions about the hydration plan to follow to achieve good hydration status until the hour of the training or the match.

On the same questionnaire, they were asked to fulfil the wellness questionnaire. This questionnaire includes questions about stress, fatigue, sleep, mood, and muscle soreness, and it is well accepted as an indicator of the well-being status of the athlete [24, 29].

**Figure 1:**
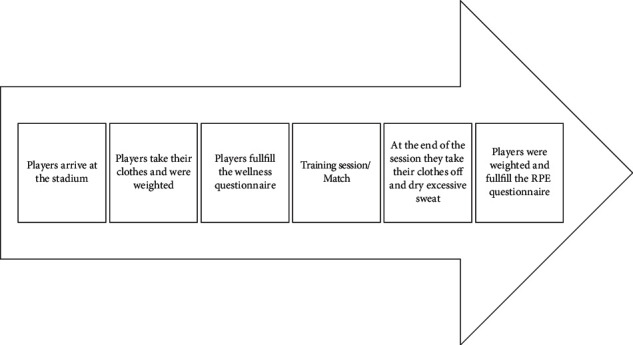
Schematic of the timing of the procedures with the players.

Furthermore, after the training session or match, players were asked to register their Rating of Perceived Exertion (RPE) in a questionnaire prepared for this purpose, using the scale CR-10 proposed by Borg [30]. This value was then multiplied by the duration of the session in order to attain the internal load [31]. The schematic of the procedures players followed is represented in Figure 1.

In every session, players utilize a harness in the upper back with a portable global positioning system (GPS) device operating at a sampling frequency of 1 kHz provided at 100 Hz that allowed the activity to be recorded (Vector device, Catapult Sports), which is already proven valid, reliable, and time-effective equipment [32]. The system uses signals from at least three earth-orbiting satellites to determine the position and calculate movement speeds and distances. After recording, the data were downloaded to a PC and analyzed using the software package. The data analyzed were the total distance covered (TDC), the player load (PL), ACC, DCC, run of high intensity (RHI), sprint (SP), and maximal sprint (_max_S).

### 2.3. Statistical Procedures

For data processing, mean and standard deviation were used. Descriptive statistics were calculated for each variable. Data were not normally distributed; thus, nonparametric tests were used for analysis. The Kruskal-Wallis *H* test was used to analyze the dehydration measures (urine color (UC), start weight (SW), final weight (FW), and percentage of dehydration (D)), wellness values (fatigue (F), sleep (Sl), muscle soreness (MS), stress (S), mood (M), and Total Wellness (TW)), and training load (TL) (duration of season (Dur), RPE, internal total load (ITL), TD, player load (PL), acceleration (ACC), decelerations (DCC), run of high intensity (RHI), SP (sprint), and maximal sprint (_max_S)) in microcycle 1 (from week 1 to week 4), microcycle 2 (from week 5 to week 8), microcycle 3 (from week 9 to week 12), and microcycle 4 (from week 13 to week 16). Posteriorly, Pearson's correlation coefficient *r* was used to examine the relationship between the percentage of change of dehydration measures, wellness values, and training load: 100 − microcycle 1∗100/microcycle 4. To interpret the magnitude of these correlations, we adopted the following criteria: *r* ≤ 0.1, trivial; 0.1 < *r* ≤ 0.3, small; 0.3 < *r* ≤ 0.5, moderate; 0.5 < *r* ≤ 0.7, large; 0.7 < *r* ≤ 0.9, very large; and *r* > 0.9, almost perfect. Finally, regression analysis was used to model the prediction of the percentage of change in dehydration measures from wellness values and training load with a positive correlation. The statistical analysis was performed with statistical software (version 13.1; Statsoft, Inc., Tulsa, OK, USA). For all analyses, significance was accepted at *p* < 0.05.

## 3. Results

Descriptive statistics were calculated for each variable (Table 1). First, a Kruskal-Wallis *H* test with participants' mean dehydration measures (UC, SW, FW, and D) in microcycle 1, microcycle 2, microcycle 3, and microcycle 4 did not reveal any significant effects. Another Kruskal-Wallis *H* test with participants' mean wellness values (F and MS) in microcycle 1, microcycle 2, microcycle 3, and microcycle 4 revealed significant effects (*H* = 14.36, *p* = 0.002, and *H* = 18.24, *p* = 0.001), respectively. However, the same analysis with participants' mean wellness values (Sl, S, M, and TW) in microcycle 1, microcycle 2, microcycle 3, and microcycle 4 did not reveal any significant effects. Last, a new Kruskal-Wallis *H* test with participants' mean training load (Dur, RPE, ITL, TD, PL, RHI, and _max_S) in microcycle 1, microcycle 2, microcycle 3, and microcycle 4 showed significant effects (*H* = 19.09, *p* = 0.001; *H* = 29.44, *p* = 0.001; *H* = 34.26, *p* = 0.001; *H* = 21.03, *p* = 0.001; *H* = 17.32, *p* = 0.006; *H* = 12.86, *p* = 0.004, and *H* = 7.58, *p* = 0.05), respectively. Nevertheless, the same analysis with participants' mean training load (ACC, DCC, and SP) in microcycle 1, microcycle 2, microcycle 3, and microcycle 4 did not reveal any significant effects. See Table 1 for more information.

Correlation analyses were performed between the percentage of dehydration measures (UC, SW, FW, and D) and wellness variables (F, Sl, MS, S, M, and TW; Table 2). Thus, data showed a moderate negative correlation between the percentage of change of SW and the percentage of change of S (*r* = −0.38, *p* = 0.04). In this sense, another two small negative correlations were found between % of change of D, the percentage of change of fatigue, and the percentage of change of TW (*r* = −0.42, *p* = 0.02 and *r* = −0.45, *p* = 0.01), respectively. Crucially. a new negative moderate correlation was found between the percentage of change of D and the percentage of change of S (*r* = −0.40, *p* = 0.03).

**Figure 2:**
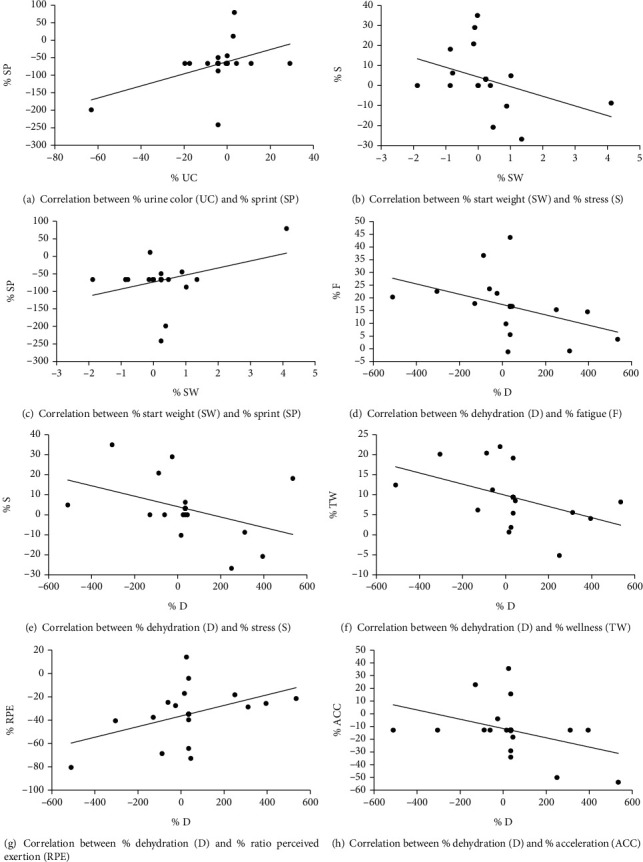
Significant correlations between dehydration measures and percentage of change of wellness variables and training load measures.

Along the same line, new correlation analysis has been performed on the percentage of dehydration measures (UC, SW, FW, and D) and training load measures (Dur, RPE, ITL, TD, PL, ACC, DCC, RHI, and SP). In consequence, data showed a moderate positive correlation between the percentage of change of UC and the percentage of change of SP (*r* = 0.46, *p* = 0.01). Complementarily, another correlation analysis between the percentage of change of SW and percentage of change of S showed a moderate positive correlation (*r* = 0.36, *p* = 0.05). Another correlation analysis with the percentage of D and percentage of change of RPE reflected a moderate positive correlation (*r* = 0.44, *p* = 0.015). The last correlation analysis with the percentage of D and percentage of change of ACC reflected a moderate negative correlation (*r* = −0.39, *p* = 0.034). See Figure 2 for more information.

Finally, a multilinear regression analysis was performed to verify which variable of dehydration measures (agreement with the correlation analysis) could be used to better explain the percentage of wellness variables (% SW and % S, *r* = −0.38; % D and % F, *r* = −0.42; % D and % S, *r* = −0.41; and % D and % TW, -0.45) and training load measures (% U and % SP, *r* = 0.46; % SW and % SP, *r* = 0.36; % D and % RPE, *r* = 0.44; and % D and % ACC, -0.39). For more information, see Table 3.

## 4. Discussion

The effect of hydration status on team sport performance has been extensively studied in soccer. Previous studies showed that significant dehydration (mean body mass loss > 2%) was detected after matches or training sessions in soccer [13, 33, 34]. Because high-intensity activities occur so frequently in a 90-minute soccer match, players' prematch hydration must be in an optimal balance. However, adequate hydration contributes to preserving physical performance and cardiovascular functions during activities in soccer [10, 24, 27]. In light of this information, our study is aimed at analyzing the relationships between fluid loss and internal and external training load in a professional soccer team throughout the first three months of a season. The present study showed a positive relationship between dehydration due to fluid loss and RPE but a negative relationship between ACCs. Moreover, our study indicated that players were not dehydrated according to urine color (values 1 to 4), which resulted in higher sprint numbers.

The urine color chart is a popular method used to predict the hydration status of soccer players before training and games because it is noninvasive, inexpensive, and reliable [27, 35, 36]. Moreover, it shows good sensitivity, specificity, and precision compared to urine-specific gravity and osmolality measures. A darker urine color (values of 5–8) indicates muscle damage and dehydration, whereas a lighter urine color (values of 1–4) (pale yellow) indicates euhydration [35–37]. The present study demonstrated that the percentage of change in urine color was positively correlated to the percentage of change in sprinting distance (>25.2 km·h^−1^). In one study, it was previously emphasized that dehydration could harm sprint performance [38]. Another study indicated that dehydration had no significant effect on the sprint activity (>22 km·h^−1^) of soccer players during a simulated game [15]. However, the above studies showed that dehydration had a negative effect on sprint performance and did not have any significant effect. In our study, we also assess urine color to determine dehydration of the athletes first thing in the morning. Contrary to these studies above, our study showed that the urine color was around 3 on average during the preseason period (4 microcycles). This result indicated to us that the players were not dehydrated according to urine color (values between 1 and 4) upon waking up. On the contrary, they were in a state of euhydration and could exhibit higher sprint distances.

High-intensity explosive eccentric movements such as accelerations and decelerations are among the critical determinants of external biomechanical load in team sports and are directly related to neuromuscular performance capacity [39]. A previous study showed that accelerations and decelerations caused 12-16% of the total player load. It shows that the load caused by accelerations and decelerations constitutes a significant part of the total load for a player during the match [40]. The present study revealed a negative correlation between the percentage of change of dehydration and the percentage of change of ACC. As dehydration (loss of body mass) increased after matches or training sessions, a decrease in the number of ACCs occurred.

Contrary to our study, a previous study showed that dehydration had no significant effect on activities lasting less than 15 seconds, and it was highlighted that this result might be related to the lactic anaerobic energy system (i.e., it does not require water) used during the specified period [41]. In other studies partially related to the results of our study, it was clearly demonstrated that dehydration after matches or training sessions harmed parameters associated with high-intensity activities [12, 24, 26, 35]. Additionally, our result supported the previous studies carried out by Djaoui et al. [42], who stated that the amount of fluid loss during official soccer games affected the numbers of high-intensity activity running distances. The same authors also argued that the amount of physical activity was not the only factor that can explain dehydration (body mass-related fluid loss) at the end of a game. It was reported that this situation might also be related to individual metabolism, aerobic fitness, and heat acclimation level [42]. Lastly, we can say that dehydration, which occurs as a result of training and matches in the preseason period, is associated with a decrease in the number of ACCs.

In the literature, the relationship between dehydration and RPE has been the focus of scientific interest [43]. The present study demonstrated a positive correlation between the percentage of change of dehydration and the percentage of change of RPE. This result is in line with previous studies showing that lower hydration status or higher fluid loss (dehydration) harmed RPE and generally triggered higher RPE values [38, 44–47]. Similarly, a recent study on professional soccer players reported that the percentage of dehydration was higher in exercises performed at RPE = 6-8 than RPE = 2-4 [47]. Contrary to the current study and the above studies, some studies reported that dehydration did not cause any significant effect on RPE values [48–50].

Furthermore, a recent systematic review with a meta-analysis study revealed that RPE increased by 0.21 points for each 1% increase in dehydration and that the effect of dehydration on RPE was unlikely to be practically significant until at least 3% body mass was lost [43]. The relationship between dehydration and RPE can be explained physiologically as follows: environmental temperature and relative humidity are the most important environmental factors that cause changes in hydration status and sweating rate and thermal discomfort. These variables strongly affect the body's thermoregulation system. For instance, the higher the environmental temperature, the greater the sweating rate. Conversely, as relative humidity increases, less sweat evaporates, which raises the athlete's body temperature; in turn, the rate of sweating increases the RPE [44].

Furthermore, perceived exertion could be considered the “mastermind” of exercise performance. High RPE due to dehydration may decrease athletes' exercise performance [13, 43]. Remarkably, recent studies showed that RPE increased significantly under dehydration conditions when compared with euhydration conditions, therefore having a detrimental effect on the physical and technical performance of rugby players [46] and soccer players [47]. Additionally, McGregor et al. [51] reported that dehydration equal to a 2.5% body mass deficit increased RPE and negatively impacted sprinting distance and soccer dribbling ability. Regarding the above studies, dehydration was observed to be associated with higher RPE values, negatively impacting performance. A previous study suggested that the decrease in performance due to dehydration is either the physiological processes that mediate the thermal strain or the negative psychological factors associated with the perception of more effort when players are dehydrated (perceived discomfort) [52]. Moreover, the available evidence from recent studies asserted that possible potential mechanisms of the detrimental effects of dehydration on team sport performance include cardiovascular strain [33], decreased plasma volume and muscle blood flow, a hypothermic effect of dehydration [13], increased core temperature and muscle glycogen use, and decreased cerebral function [15]. More studies are needed to determine whether water loss is detrimental to performance [52].

### 4.1. Limitations

The present study has some limitations. Firstly, the sample group of the study is small. It can make it difficult to generalize the results. Therefore, it is essential to increase the number of studies on male and female amateur or professional soccer players in different age categories in different countries. Second, in the present study, the players' hydration levels, internal and external load parameters, and wellness measures were followed only during the preseason period rather than the whole season. This may also be a limitation. Also, we did not use any physiological values to assess hydration, and although urine color is an accepted method to assess hydration, it is not the standard gold method. In addition, the players' nutritional status or food consumption records were not tracked during the study period. It may be the third limitation. We also need to recognize that the questionnaires used, although validated for use with athletes, do not have validation for the Portuguese language. Finally, in our study, player positions were not taken into account. In soccer, the physical demands of player positions during a match (e.g., high-intensity running and sprinting) vary, and fluid losses may change accordingly [12, 42, 53]. For this reason, it is recommended to carry out longitudinal studies considering the player position in the future.

## 5. Conclusion

The present study revealed significant differences in the wellness and training load parameters between microcycles during the study period. Furthermore, our study showed a moderate positive correlation between the percentage of change in urine color and the percentage change in the sprint distance. Since the athletes in our study were not dehydrated according to urine color (values between 1 and 3), it was expected they could exhibit high sprint distances. The point is that dehydration can increase the perceived effort but also harms the number of accelerations. In that regard, it is crucial to manage the degree of dehydration of the players to allow performance to be the best. This result showed that dehydration after the preseason period increased the perceived physical exertion and reduced the number of ACCs in soccer players. Therefore, considering the negative effect of dehydration on physical exertion and ACCs, keeping the hydration status of the players at an optimal level during the whole season before training and matches can contribute to the display of a high physical activity profile during matches and minimize the possible risk of injury.

Even with the limitations observed, our study can provide some insights into the hydration intervention in soccer players. With the knowledge of the impact, dehydration was in some of the performance variables, and the practical application of hydration strategies to minimize this impact can be significant throughout the season.

## Figures and Tables

**Table 1:** Dehydration, wellness, and training load variables in microcycle 1, microcycle 2, microcycle 3, and microcycle 4.

	Microcycle 1	Microcycle 2	Microcycle 3	Microcycle 4	%	Kruskal-Wallis *H*
*Dehydration measures*						
UC (A.U.)	3.26 ± 0.89	3.22 ± 0.76	3.22 ± 1.09	3.09 ± 0.74	-4.23	*H* = 0.80|*p* = 0.84
SW (kg)	77.66 ± 8.32	76.23 ± 8.11	76.95 ± 8.02	78.16 ± 8.05	0.23	*H* = 1.01|*p* = 0.79
FW (kg)	77.11 ± 8.39	76.03 ± 8.23	77.10 ± 7.79	77.23 ± 8.36	0.36	*H* = 0.48|*p* = 0.92
D (%)	0.01 ± 0.01	0.01 ± 0.01	−0.03 ± 0.07	0.01 ± 0.01	35.22	*H* = 1.93|*p* = 0.58

*Wellness*						
F (A.U.)	2.94 ± 0.63	3.14 ± 0.43	3.29 ± 0.49	3.53 ± 0.54	16.67	*H* = 14.36|*p* = 0.002^∗∗^
Sl (A.U.)	3.82 ± 0.55	3.78 ± 0.35	3.82 ± 0.27	3.91 ± 0.44	3.82	*H* = 2.68|*p* = 0.44
MS (A.U.)	2.71 ± 0.64	3.26 ± 0.51	3.39 ± 0.56	3.53 ± 0.59	24.33	*H* = 18.24|*p* = 0.001^∗∗^
S (A.U.)	3.64 ± 0.66	3.79 ± 0.56	3.73 ± 0.55	3.83 ± 0.60	3.17	*H* = 1.49|*p* = 0.68
M (A.U.)	4.11 ± 0.53	4.08 ± 0.60	4.04 ± 0.47	4.03 ± 0.49	-1.54	*H* = 0.51|*p* = 0.91
TW (A.U.)	17.28 ± 2.48	17.74 ± 2.26	18.25 ± 1.52	18.83 ± 2.25	9.38	*H* = 6.25|*p* = 0.09

*Training load*						
Dur (min)	90.21 ± 16.2	69.95 ± 9.93	67.97 ± 14.05	71.50 ± 7.72	-31.69	*H* = 19.09|*p* = 0.001^∗∗^
RPE (Borg)	6.89 ± 0.78	5.75 ± 0.82	5.67 ± 0.97	5.20 ± 1.01	-34.85	*H* = 29.44|*p* = 0.001^∗∗^
ITL (AU)	782.55 ± 138.15	499.80 ± 97.84	428.37 ± 100.45	405.31 ± 84.04	-117.37	*H* = 34.26|*p* = 0.001^∗∗^
TD (m)	7843.71 ± 1348.24	6240.77 ± 1454.12	5333.01 ± 1378.38	5457.11 ± 772.95	-48.96	*H* = 21.03|*p* = 0.001^∗∗^
PL (GPS)	818.14 ± 165.36	654.27 ± 141.69	578.59 ± 144.52	591.79 ± 94.74	-44.02	*H* = 17.32|*p* = 0.006^∗∗^
ACC (num)	19.48 ± 5.57	19.37 ± 6.36	16.67 ± 4.71	19.44 ± 6.49	-12.85	*H* = 3.60|*p* = 0.30
DCC (num)	23.71 ± 9.12	24.39 ± 8.41	19.76 ± 6.78	20.34 ± 5.95	-22.22	*H* = 5.76|*p* = 0.12
RHI (m)	294.24 ± 159.04	282.83 ± 96.82	196.07 ± 86.17	210.27 ± 72.11	-43.87	*H* = 12.86|*p* = 0.004^∗∗^
SP (m)	97.85 ± 93.28	61.72 ± 38.74	40.91 ± 25.51	52.90 ± 27.98	-66.27	*H* = 5.44| *p* = 0.14
_max_S (km·h^−1^)	29.21 ± 3.67	27.73 ± 1.42	27.02 ± 1.09	27.49 ± 1.67	-6.41	*H* = 7.58|*p* = 0.05^∗^

Note: %: percentage of change; UC: urine color; SW: start weight; FW: final weight; D: dehydration: F: fatigue; Sl: sleep; MS: muscle soreness; S: stress; M: mood; TW: total wellness; Dur: duration of session; RPE: rating perceived exertion; ITL: total load; TD: total distance; PL: player load; ACC: acceleration; DCC: deceleration; RHI: run of high intensity (>25.2); SP: sprint (>25.2); _max_S: maximal sprint; A.U.: arbitrary units. ^∗^ denotes significance at *p* < 0.05, and ∗∗ denotes significance at *p* < 0.01.

**Table 2:** Correlation between percentage of dehydration measures (UC, SW, FW, and D) and percentage of change of wellness variables (F, Sl, MS, S, M, and TW) and training load measures (Dur, RPE, ITL, TD, PL, ACC, DCC, RHI, SP, and _max_S).

		% F	% Sl	% MS	% S	% M	% TW	% Dur	% RPE	% ITL	% TD	% PL	% ACC	% DCC	% RHI	% SP	% _max_S
% UC	*r*	-0.01	-0.02	0.13	0.30	-0.17	0.12	-0.01	-0.08	0.03	-0.01	-0.10	-0.11	-0.11	0.23	0.46	0.14
	*p*	0.92	0.90	0.47	0.10	0.35	0.52	0.93	0.66	0.83	0.93	0.57	0.55	0.56	0.21	0.01^∗^	0.45
	L95%	-0.42	-0.43	-0.26	-0.08	-0.57	-0.28	-0.47	-0.48	-0.36	-0.42	-0.52	-0.51	-0.52	-0.16	-0.11	-0.25
	U95%	0.39	0.38	0.54	0.69	0.22	0.52	0.41	0.32	0.44	0.39	0.26	0.29	0.29	0.63	0.82	0.54

% SW	*r*	-0.30	-0.16	0.21	-0.38	-0.32	-0.28	-0.15	0.11	0.01	-0.03	-0.16	-0.06	-0.09	0.18	0.36	0.33
	*p*	0.10	0.39	0.26	0.04^∗^	0.08	0.14	0.42	0.55	0.93	0.86	0.38	0.75	0.63	0.32	0.05^∗^	0.08
	L95%	-0.69	-0.56	-0.18	-0.76	-0.71	-0.67	-0.69	-0.29	-0.39	-0.44	-0.56	-0.46	-0.49	-0.21	-0.01	-0.05
	U95%	0.08	0.23	0.61	0.01	0.06	0.11	0.16	0.51	0.42	0.37	0.23	0.34	0.31	0.58	0.74	0.71

% FW	*r*	-0.08	-0.03	0.13	-0.18	-0.26	-0.07	-0.12	-00.05	0.03	0.04	-0.14	-0.11	-0.05	0.20	0.25	0.34
	*p*	0.67	0.85	0.48	0.33	0.16	0.71	0.52	0.78	0.87	0.82	0.43	0.53	0.77	0.29	0.18	0.06
	L95%	-0.48	-0.44	-0.26	-0.58	-0.65	-0.47	-0.62	-0.46	-0.37	-0.36	-0.55	-0.52	-0.46	-0.19	-0.14	-0.03
	U95%	0.32	0.37	0.54	0.21	0.12	0.33	0.25	0.35	0.43	0.45	0.25	0.28	0.35	0.60	0.64	0.73

% D	*r*	-0.42	-0.35	-0.02	-0.40	-0.07	-0.45	0.05	0.44	0.11	-0.04	-0.02	-0.39	-0.24	0.021	0.21	-0.12
	*p*	0.02^∗^	0.05	0.90	0.03^∗^	0.70	0.01^∗^	0.78	0.01^∗^	0.54	0.82	0.91	0.03^∗^	0.19	0.91	0.25	0.52
	L95%	-0.79	-0.73	-0.43	-0.77	-0.48	-0.81	-0.37	-0.09	-0.28	-0.45	-0.42	-0.76	-0.64	-0.38	-0.18	-0.52
	U95%	0.06	0.02	0.38	-0.03	0.33	0.09	0.51	0.81	0.52	0.36	0.38	0.02	0.14	0.42	0.61	0.28

Note: U: urine color; SW: start weight; FW: final weight; D: dehydration: F: fatigue; Sl: sleep; MS: muscle soreness; S: stress; M: mood; TW: total wellness; Dur: duration of session; RPE: rating perceived exertion; ITL: internal total load; TD: total distance; PL: player load; ACC: acceleration; DCC: deceleration; RHI: run of high intensity (>25.2); SP: sprint (>25.2); _max_S: maximal sprint. ∗ denotes significance at *p* < 0.05, and ∗∗ denotes significance at *p* < 0.

**(a):** 

Dehydration measures	Wellness variables	*β* ^∗^	Standard *β*^∗^	*R*	*R* ^2^	Adjusted *R*^2^	*F*	*p*
% SW	% S	-0.38	0.18	0.38	0.14	0.11	4.65	0.03^∗^
% D	% F	-0.42	0.17	0.42	0.17	0.14	5.87	0.02^∗^
% D	% S	-0.41	0.18	0.40	0.16	0.13	5.20	0.03^∗^
% D	% TW	0.48	0.17	0.45	0.20	0.17	6.97	0.01^∗^

**(b):** 

Dehydration measures	Training load measures	*β* ^∗^	Standard *β*^∗^	*R*	*R* ^2^	Adjusted *R*^2^	*F*	*p*
% UC	% SP	0.46	0.17	0.46	0.21	0.18	7.42	0.01^∗^
% SW	% SP	0.36	0.18	0.36	0.15	0.10	4.22	0.05^∗^
% D	% RPE	-44	0.17	0.44	0.20	0.17	6.77	0.01^∗^
% D	% ACC	-0.39	0.18	0.39	0.15	0.12	4.96	0.03^∗^

Note: U: urine color; SW: start weight; D: dehydration: F: fatigue; S: stress; TW: total wellness; RPE: rating perceived exertion; ACC: acceleration; SP: sprint (>25.2); ∗ denotes significance at *p* < 0.05, and ∗∗ denotes significance at *p* < 0.01.

## Data Availability

All data generated or analyzed during this study are included in this published article and their supplementary information files.
