# Supplementary material for: Dehydration, Wellness, and Training Demands of Professional Soccer Players during Preseason
Source: Biomed Res Int. 2022 Nov 7;2022:8054449. doi: 10.1155/2022/8054449 (PMC9663222; doi:10.1155/2022/8054449)
Supplement: Supplementary Materials — The data of this study are available in this file. [file 8054449.f1.pdf]

UC	Microcycle
2/92	1/00
4/08	1/00
3/29	1/00
2/85	1/00
	1/00
2/06	1/00
3/56	1/00
	1/00
3/02	1/00
2/90	1/00
2/12	1/00
5/80	1/00
4/00	1/00
3/08	1/00
	1/00
3/00	1/00
4/20	1/00
	1/00
2/00	1/00
3/12	1/00
3/00	1/00
4/00	1/00
	1/00
	1/00
	1/00
3/00	1/00
	1/00
	1/00
2/94	2/00
4/18	2/00
3/37	2/00
3/53	2/00
	2/00
1/95	2/00
3/25	2/00
3/35	2/00
3/29	2/00
2/99	2/00
2/10	2/00
5/00	2/00
	2/00
3/15	2/00
3/95	2/00
3/04	2/00
2/63	2/00
3/96	2/00

M1	M2	M3
3/26	3/22	3/22
0/89	0/76	1/09

Kruskal-Wallis ANOVA by
Independent (group
Kruskal-Wallis test: H (3, N

	Code	Valid
1	1	19
2	2	25
3	3	24
4	4	24

2/00	2/00
3/06	2/00
3/05	2/00
4/00	2/00
2/25	2/00
3/07	2/00
4/54	2/00
2/78	2/00
	2/00
3/06	2/00
2/96	3/00
4/15	3/00
	3/00
3/79	3/00
2/50	3/00
1/84	3/00
3/00	3/00
2/96	3/00
3/22	3/00
2/56	3/00
2/13	3/00
	3/00
	3/00
3/38	3/00
4/27	3/00
3/06	3/00
2/45	3/00
4/00	3/00
2/00	3/00
3/08	3/00
3/00	3/00
6/94	3/00
2/50	3/00
3/00	3/00
5/00	3/00
2/58	3/00
	3/00
3/00	3/00
3/00	4/00
3/41	4/00
	4/00
4/02	4/00
2/00	4/00
1/75	4/00
	4/00
3/11	4/00
3/00	4/00

3/00	4/00
2/39	4/00
	4/00
	4/00
3/22	4/00
4/25	4/00
3/00	4/00
2/58	4/00
4/00	4/00
2/00	4/00
3/40	4/00
3/00	4/00
4/00	4/00
2/25	4/00
3/00	4/00
4/75	4/00
2/75	4/00
3/33	4/00
3/00	4/00

M4
3/09
0/74

' Ranks; Var1 (Spreadsheet1)
)ing) variable: Var2
N= 92) =,8039039 p =,8485

Sum of	Mean
915/000	48/15789
1242/000	49/68000
1050/000	43/75000
1071/000	44/62500

SW	Microcycle
67/53	1/00
83/58	1/00
64/93	1/00
85/00	1/00
	1/00
94/09	1/00
66/00	1/00
	1/00
79/63	1/00
78/47	1/00
84/94	1/00
77/72	1/00
92/29	1/00
79/98	1/00
	1/00
72/72	1/00
69/51	1/00
	1/00
82/16	1/00
78/57	1/00
69/00	1/00
76/37	1/00
	1/00
	1/00
	1/00
72/95	1/00
	1/00
	1/00
67/16	2/00
83/75	2/00
64/50	2/00
85/07	2/00
	2/00
93/40	2/00
66/13	2/00
95/63	2/00
77/56	2/00
80/06	2/00
84/90	2/00
77/54	2/00
	2/00
80/31	2/00
72/90	2/00
72/00	2/00
69/31	2/00
69/44	2/00

**82/04**	2/00
**79/35**	2/00
**69/00**	2/00
**76/48**	2/00
**72/35**	2/00
**75/59**	2/00
**68/86**	2/00
**72/35**	2/00
	2/00
**70/12**	2/00
**67/43**	3/00
**83/18**	3/00
	3/00
**84/96**	3/00
**83/00**	3/00
**92/79**	3/00
**67/00**	3/00
**95/65**	3/00
**78/33**	3/00
**80/65**	3/00
**84/92**	3/00
	3/00
	3/00
**80/35**	3/00
**70/00**	3/00
**72/00**	3/00
**69/45**	3/00
**69/43**	3/00
**82/25**	3/00
**79/96**	3/00
**69/00**	3/00
**78/17**	3/00
**72/25**	3/00
**75/97**	3/00
**68/40**	3/00
**71/46**	3/00
	3/00
**70/17**	3/00
**67/45**	4/00
**83/97**	4/00
	4/00
**84/98**	4/00
**83/00**	4/00
**92/35**	4/00
	4/00
**96/57**	4/00
**79/00**	4/00

**81/84**	4/00
**86/10**	4/00
	4/00
	4/00
**79/87**	4/00
**72/93**	4/00
**72/10**	4/00
**69/77**	4/00
**69/30**	4/00
**82/15**	4/00
**79/38**	4/00
**69/00**	4/00
**77/05**	4/00
**72/59**	4/00
**76/00**	4/00
**69/08**	4/00
**72/33**	4/00
**89/08**	4/00
**70/05**	4/00

M1	M2	M3	M4
77/66	76/23	76/95	78/16
8/32	8/11	8/02	8/05

Kruskal-Wallis ANOVA by Ranks; Var1 (Spreadsheet1)
Independent (grouping) variable: Var2
Kruskal-Wallis test: H (3, N= 92) =1,005591 p =,7999

	Code	Valid	Sum of	Mean
1	1	19	915/500	48/18421
2	2	25	1071/000	42/84000
3	3	24	1090/500	45/43750
4	4	24	1201/000	50/04167

FW	Microcycle
**66/58**	1/00
**83/74**	1/00
**64/00**	1/00
**84/63**	1/00
	1/00
**93/23**	1/00
**66/00**	1/00
	1/00
**79/16**	1/00
**78/64**	1/00
**84/83**	1/00
**77/14**	1/00
**92/02**	1/00
**78/96**	1/00
	1/00
**72/44**	1/00
**68/60**	1/00
	1/00
**81/47**	1/00
**77/00**	1/00
**69/00**	1/00
**75/85**	1/00
	1/00
	1/00
	1/00
**71/75**	1/00
	1/00
	1/00
**66/48**	2/00
**83/49**	2/00
**64/47**	2/00
**84/78**	2/00
	2/00
**92/00**	2/00
**66/00**	2/00
**95/72**	2/00
**77/50**	2/00
**80/22**	2/00
**85/00**	2/00
**77/25**	2/00
	2/00
**79/35**	2/00
**72/33**	2/00
**72/00**	2/00
**68/86**	2/00
**69/10**	2/00

M1	M2	M3
**77/11**	**76/03**	**77/10**
**8/39**	**8/23**	**7/79**

Kruskal-Wallis ANOVA by Ranks; Var1 (S
Independent (grouping) variable: V
Kruskal-Wallis test: H (3, N= 89) =,48200

Code	Valid	Sum of
1	19	854/500
2	24	1009/000
3	23	1068/500
4	23	1073/000

**81/77**	2/00
**79/13**	2/00
**69/00**	2/00
**75/91**	2/00
	2/00
**75/41**	2/00
**68/47**	2/00
**71/16**	2/00
	2/00
**69/43**	2/00
**66/58**	3/00
**82/86**	3/00
	3/00
**84/71**	3/00
**83/00**	3/00
**91/72**	3/00
	3/00
**95/37**	3/00
**78/33**	3/00
**80/67**	3/00
**85/13**	3/00
	3/00
	3/00
**79/41**	3/00
**71/92**	3/00
**72/00**	3/00
**69/08**	3/00
**68/98**	3/00
**81/91**	3/00
**79/17**	3/00
**69/00**	3/00
**75/99**	3/00
**73/00**	3/00
**75/75**	3/00
**69/00**	3/00
**70/28**	3/00
	3/00
**69/37**	3/00
**66/75**	4/00
**83/89**	4/00
	4/00
**84/92**	4/00
	4/00
**90/42**	4/00
**66/00**	4/00
**95/44**	4/00
**78/85**	4/00

**81/62**	4/00
**86/11**	4/00
	4/00
	4/00
**79/18**	4/00
**72/15**	4/00
**72/20**	4/00
**69/21**	4/00
**68/87**	4/00
**81/86**	4/00
**79/24**	4/00
**69/00**	4/00
**76/44**	4/00
	4/00
**76/00**	4/00
**69/00**	4/00
**70/61**	4/00
**88/95**	4/00
**69/68**	4/00

M4
77/23
8/36

Spreadsheet1)
Var2
...82 p =,9228

Mean
44/97368
42/04167
46/45652
46/65217

D	Microcycle
0/01	1/00
0/00	1/00
0/01	1/00
0/00	1/00
	1/00
0/01	1/00
0/00	1/00
	1/00
0/01	1/00
0/00	1/00
0/00	1/00
0/01	1/00
0/00	1/00
0/01	1/00
	1/00
0/01	1/00
0/01	1/00
	1/00
0/01	1/00
0/02	1/00
0/00	1/00
0/01	1/00
	1/00
	1/00
	1/00
0/02	1/00
	1/00
	1/00
0/01	2/00
0/00	2/00
0/00	2/00
0/00	2/00
	2/00
0/01	2/00
0/00	2/00
0/00	2/00
0/00	2/00
0/00	2/00
0/00	2/00
0/00	2/00
	2/00
0/01	2/00
0/01	2/00
0/00	2/00
0/01	2/00
0/01	2/00

M1
0/01
0/01

1
2
3
4

**0/01**	2/00
**0/00**	2/00
**0/00**	2/00
**0/01**	2/00
	2/00
**0/00**	2/00
**0/01**	2/00
**0/02**	2/00
	2/00
**0/01**	2/00
**0/01**	3/00
**0/00**	3/00
	3/00
**0/00**	3/00
**0/00**	3/00
**0/01**	3/00
	3/00
**0/00**	3/00
**0/00**	3/00
**0/00**	3/00
**0/00**	3/00
	3/00
	3/00
**0/01**	3/00
**-0/70**	3/00
**0/00**	3/00
**0/01**	3/00
**0/01**	3/00
**0/00**	3/00
**0/01**	3/00
**0/00**	3/00
**0/02**	3/00
	3/00
**0/00**	3/00
**-0/01**	3/00
**0/02**	3/00
	3/00
**0/01**	3/00
**0/01**	4/00
**0/00**	4/00
	4/00
**0/00**	4/00
	4/00
**0/02**	4/00
	4/00
**0/00**	4/00
**0/00**	4/00

**0/00**	4/00
**0/00**	4/00
	4/00
	4/00
**0/01**	4/00
**0/01**	4/00
**0/00**	4/00
**0/01**	4/00
**0/01**	4/00
**0/00**	4/00
**0/00**	4/00
**0/00**	4/00
**0/01**	4/00
	4/00
**0/00**	4/00
**0/00**	4/00
**0/02**	4/00
**0/00**	4/00
**0/00**	4/00

M2	M3	M4
0/01	-0/03	0/01
0/01	0/15	0/01

Kruskal-Wallis ANOVA by Ranks; Var1 (Spreadsheet1)
Independent (grouping) variable: Var2
Kruskal-Wallis test: H (3, N= 87) =1,938919 p =,5852

Code	Valid	Sum of	Mean
1	19	968/000	50/94737
2	24	1036/000	43/16667
3	22	924/000	42/00000
4	22	900/000	40/90909

F
2/44
3/19
4/61
3/05
2/50
2/56
2/25
3/28
2/43
2/20
4/00
2/53
2/61
2/73
3/29
2/72
2/83
3/81
2/83
2/44
3/28
3/77
2/98
2/91
3/54
2/75
3/18
2/69
2/98
2/83
3/71
3/38
2/69
3/00
3/96

3/60
2/75
3/05
3/95
2/83
3/50
3/25
3/06

2/56
3/33
3/56

2/79
3/25
2/83
4/00
2/90
2/78
2/81
2/60

4/00
3/94
3/00
3/77
3/80
3/89
3/21
3/00
4/06
3/00
3/73
3/00
2/69

3/00
3/13
3/74

3/94
4/00
3/00

3/45
4/00

3/25
2/88

4/00
4/05
2/71
3/58
4/00
4/00
3/42
3/00
4/23
3/00
3/83
4/67
2/80
3/00
3/00

Microcycle
1/00
1/00
1/00
1/00
1/00
1/00
1/00
1/00
1/00
1/00
1/00
1/00
1/00
1/00
1/00
1/00
1/00
1/00
1/00
1/00
1/00
1/00
1/00
1/00
1/00
1/00
1/00
1/00
2/00
2/00
2/00
2/00
2/00
2/00
2/00
2/00
2/00
2/00
2/00
2/00
2/00
2/00
2/00
2/00
2/00
2/00

M1	M2	M3	M4
2/94	3/14	3/29	3/53
0/63	0/43	0/49	0/54

Kruskal-Wallis ANOVA by Ranks; Var1 (Spreads
Independent (grouping) variable: Var2
Kruskal-Wallis test: H (3, N= 92) =14,36882 p =,

	Code	Valid	Sum of
1	1	19	583/000
2	2	25	1057/500
3	3	24	1180/000
4	4	24	1457/500

2/00
2/00
2/00
2/00
2/00
2/00
2/00
2/00
2/00
2/00
3/00
3/00
3/00
3/00
3/00
3/00
3/00
3/00
3/00
3/00
3/00
3/00
3/00
3/00
3/00
3/00
3/00
3/00
3/00
3/00
3/00
3/00
3/00
3/00
3/00
3/00
3/00
3/00
4/00
4/00
4/00
4/00
4/00
4/00
4/00
4/00
4/00

4/00
4/00
4/00
4/00
4/00
4/00
4/00
4/00
4/00
4/00
4/00
4/00
4/00
4/00
4/00
4/00
4/00
4/00
4/00

heet1)

0024

Mean
30/68421
42/30000
49/16667
60/72917

SI	Microcycle
2/61	1/00
3/94	1/00
4/71	1/00
4/05	1/00
	1/00
4/00	1/00
2/83	1/00
	1/00
3/92	1/00
3/97	1/00
3/80	1/00
4/80	1/00
4/00	1/00
3/65	1/00
	1/00
3/17	1/00
3/20	1/00
	1/00
4/26	1/00
3/67	1/00
3/94	1/00
4/11	1/00
	1/00
	1/00
	1/00
4/00	1/00
	1/00
	1/00
2/81	2/00
3/56	2/00
3/83	2/00
4/34	2/00
	2/00
4/04	2/00
3/92	2/00
3/75	2/00
4/00	2/00
3/83	2/00
3/67	2/00
3/64	2/00
	2/00
3/77	2/00
4/13	2/00
3/21	2/00
3/08	2/00
4/00	2/00

M1
3/82
0/55

Krusl

Krus

1
2
3
4

**4/14**	2/00
**3/79**	2/00
**4/00**	2/00
**4/02**	2/00
**3/75**	2/00
**4/20**	2/00
**3/67**	2/00
**3/70**	2/00
	2/00
**3/58**	2/00
**3/80**	3/00
**3/77**	3/00
	3/00
**4/46**	3/00
**3/50**	3/00
**3/92**	3/00
**4/00**	3/00
**3/58**	3/00
**3/83**	3/00
**3/76**	3/00
**3/96**	3/00
	3/00
	3/00
**4/00**	3/00
**3/94**	3/00
**3/33**	3/00
**3/98**	3/00
**3/88**	3/00
**4/22**	3/00
**3/67**	3/00
**3/75**	3/00
**3/88**	3/00
**3/25**	3/00
**3/88**	3/00
**3/65**	3/00
**3/60**	3/00
	3/00
**4/10**	3/00
**3/28**	4/00
**4/10**	4/00
	4/00
**4/88**	4/00
**4/00**	4/00
**4/00**	4/00
	4/00
**4/00**	4/00
**4/00**	4/00

**3/90**	4/00
**3/70**	4/00
	4/00
	4/00
**4/00**	4/00
**4/05**	4/00
**3/15**	4/00
**3/64**	4/00
**4/00**	4/00
**4/13**	4/00
**3/73**	4/00
**4/00**	4/00
**4/05**	4/00
**3/50**	4/00
**3/93**	4/00
**4/92**	4/00
**4/00**	4/00
**2/92**	4/00
**4/00**	4/00

M2	M3	M4
3/78	3/82	3/91
0/35	0/27	0/44

kal-Wallis ANOVA by Ranks; Var1 (Spreadsheet1)
Independent (grouping) variable: Var2
kal-Wallis test: H (3, N= 92) =2,688270 p =,4422

Code	Valid	Sum of	Mean
1	19	918/500	48/34211
2	25	1078/500	43/14000
3	24	1006/000	41/91667
4	24	1275/000	53/12500

MS
2/03
3/11
4/20
2/70
2/28
2/00
2/25
2/42
2/31
1/80
4/00
2/57
3/00
2/68
3/28
2/67
2/31
3/29
2/58
2/31
3/62
4/00
3/29
2/73
3/63
2/56
3/23
3/56
2/98
2/75
3/85
3/75
2/78
3/06
4/00

3/70
2/77
3/05
4/31
3/00
3/35
3/54
2/98

2/78
3/62
3/77

2/83
3/00
2/85
4/00
2/90
2/78
3/68
2/73

3/92
4/02
3/51
3/96
3/88
4/12
3/54
2/67
4/31
3/00
3/73
3/25
2/79

2/40
3/05
3/89

3/79
4/00
3/00

3/53
4/00

3/95
2/79
4/00
4/00
3/39
3/58
4/00
4/00
3/01
3/00
4/36
3/00
3/78
4/67
2/85
2/50
2/65

Microcycle
1/00
1/00
1/00
1/00
1/00
1/00
1/00
1/00
1/00
1/00
1/00
1/00
1/00
1/00
1/00
1/00
1/00
1/00
1/00
1/00
1/00
1/00
1/00
1/00
1/00
1/00
1/00
1/00
2/00
2/00
2/00
2/00
2/00
2/00
2/00
2/00
2/00
2/00
2/00
2/00
2/00
2/00
2/00
2/00
2/00
2/00

M1	M2	M3	M4
2/71	3/26	3/39	3/53
0/64	0/51	0/56	0/59

Kruskal-Wallis ANOVA by Ranks; Var1 (Spreads
Independent (grouping) variable: Var2
Kruskal-Wallis test: H (3, N= 92) =18,24612 p =,

	Code	Valid	Sum of
1	1	19	470/000
2	2	25	1167/500
3	3	24	1242/000
4	4	24	1398/500

2/00
2/00
2/00
2/00
2/00
2/00
2/00
2/00
2/00
2/00
3/00
3/00
3/00
3/00
3/00
3/00
3/00
3/00
3/00
3/00
3/00
3/00
3/00
3/00
3/00
3/00
3/00
3/00
3/00
3/00
3/00
3/00
3/00
3/00
3/00
3/00
3/00
3/00
4/00
4/00
4/00
4/00
4/00
4/00
4/00
4/00
4/00

4/00
4/00
4/00
4/00
4/00
4/00
4/00
4/00
4/00
4/00
4/00
4/00
4/00
4/00
4/00
4/00
4/00
4/00
4/00

heet1)	
0004	
	Mean
	24/73684
	46/70000
	51/75000
	58/27083

S	Microcycle
**2/36**	1/00
**4/83**	1/00
**4/70**	1/00
**3/25**	1/00
	1/00
**3/00**	1/00
**3/33**	1/00
	1/00
**3/75**	1/00
**4/35**	1/00
**3/87**	1/00
**3/20**	1/00
**4/00**	1/00
**3/17**	1/00
	1/00
**3/22**	1/00
**4/00**	1/00
	1/00
**4/00**	1/00
**3/68**	1/00
**3/00**	1/00
**4/41**	1/00
	1/00
	1/00
	1/00
**3/00**	1/00
	1/00
	1/00
**2/81**	2/00
**4/00**	2/00
**4/10**	2/00
**4/88**	2/00
	2/00
**3/04**	2/00
**2/75**	2/00
**3/88**	2/00
**4/00**	2/00
**3/93**	2/00
**3/56**	2/00
**3/00**	2/00
	2/00
**3/85**	2/00
**4/00**	2/00
**3/91**	2/00
**4/00**	2/00
**3/81**	2/00

M1
**3/64**
**0/66**

Krusl
Krusl
1
2
3
4

**4/00**	2/00
**3/85**	2/00
**3/15**	2/00
**4/00**	2/00
**4/75**	2/00
**4/53**	2/00
**4/25**	2/00
**3/00**	2/00
	2/00
**3/58**	2/00
**3/36**	3/00
**3/88**	3/00
	3/00
**4/92**	3/00
**4/00**	3/00
**3/00**	3/00
**3/00**	3/00
**3/79**	3/00
**4/00**	3/00
**3/92**	3/00
**2/88**	3/00
	3/00
	3/00
**3/92**	3/00
**4/15**	3/00
**3/72**	3/00
**4/00**	3/00
**3/00**	3/00
**3/88**	3/00
**3/83**	3/00
**2/83**	3/00
**4/13**	3/00
**4/75**	3/00
**3/88**	3/00
**3/75**	3/00
**3/00**	3/00
	3/00
**4/05**	3/00
**3/33**	4/00
**4/00**	4/00
	4/00
**5/00**	4/00
**5/00**	4/00
**3/00**	4/00
	4/00
**4/00**	4/00
**4/00**	4/00

**4/00**	4/00
**3/05**	4/00
	4/00
	4/00
**4/00**	4/00
**4/00**	4/00
**3/94**	4/00
**4/00**	4/00
**3/00**	4/00
**4/00**	4/00
**3/87**	4/00
**3/00**	4/00
**4/00**	4/00
**3/75**	4/00
**4/11**	4/00
**4/83**	4/00
**3/00**	4/00
**3/08**	4/00
**4/00**	4/00

M2	M3	M4
3/79	3/73	3/83
0/56	0/55	0/60

kal-Wallis ANOVA by Ranks; Var1 (Spreadsheet1)
Independent (grouping) variable: Var2
kal-Wallis test: H (3, N= 92) =1,491355 p =,6843

Code	Valid	Sum of	Mean
1	19	798/000	42/00000
2	25	1189/000	47/56000
3	24	1063/500	44/31250
4	24	1227/500	51/14583

M
3/17
4/75
4/78
5/00
3/78
4/33
4/00
4/92
3/93
3/40
4/00
4/00
3/78
4/07
4/00
3/73
4/00
4/88
3/58
2/50
4/65
4/40
5/00
3/68
3/42
4/19
4/00
4/89
3/42
2/97
4/00
4/00
4/00
4/00
4/00

4/00
3/92
4/00
4/58
4/92
4/83
4/25
3/77

4/53
3/57
4/54

5/00
4/00
3/74
4/50
3/75
4/00
4/88
2/73

4/00
4/08
4/00
4/00
4/00
3/88
3/83
3/92
4/23
4/75
3/96
3/85
3/69

4/10
3/40
4/95

5/00
4/00
4/00

3/80
4/00

4/95
3/13

4/00
4/00
4/00
4/00
4/00
3/94
3/87
4/00
4/00
3/81
4/11
4/92
3/65
3/25
4/00

Microcycle
1/00
1/00
1/00
1/00
1/00
1/00
1/00
1/00
1/00
1/00
1/00
1/00
1/00
1/00
1/00
1/00
1/00
1/00
1/00
1/00
1/00
1/00
1/00
1/00
1/00
1/00
1/00
1/00
2/00
2/00
2/00
2/00
2/00
2/00
2/00
2/00
2/00
2/00
2/00
2/00
2/00
2/00
2/00
2/00
2/00
2/00

M1	M2	M3	M4
4/11	4/08	4/04	4/03
0/53	0/60	0/47	0/49

Kruskal-Wallis ANOVA by Ranks; Var1 (Spreads
Independent (grouping) variable: Var2
Kruskal-Wallis test: H (3, N= 92) =,5198569 p =,

	Code	Valid	Sum of
1	1	19	894/500
2	2	25	1234/500
3	3	24	1077/000
4	4	24	1072/000

2/00
2/00
2/00
2/00
2/00
2/00
2/00
2/00
2/00
2/00
3/00
3/00
3/00
3/00
3/00
3/00
3/00
3/00
3/00
3/00
3/00
3/00
3/00
3/00
3/00
3/00
3/00
3/00
3/00
3/00
3/00
3/00
3/00
3/00
3/00
3/00
3/00
3/00
4/00
4/00
4/00
4/00
4/00
4/00
4/00
4/00
4/00

4/00
4/00
4/00
4/00
4/00
4/00
4/00
4/00
4/00
4/00
4/00
4/00
4/00
4/00
4/00
4/00
4/00
4/00
4/00

heet1)

9145

Mean
47/07895
49/38000
44/87500
44/66667

TW	Microcycle
**12/61**	1/00
**19/83**	1/00
**23/00**	1/00
**18/05**	1/00
	1/00
**15/56**	1/00
**15/06**	1/00
	1/00
**16/17**	1/00
**18/93**	1/00
**16/34**	1/00
	1/00
**20/00**	1/00
**15/92**	1/00
	1/00
**15/78**	1/00
**16/68**	1/00
	1/00
**18/82**	1/00
**15/67**	1/00
**16/08**	1/00
**20/50**	1/00
	1/00
	1/00
	1/00
**16/00**	1/00
	1/00
	1/00
**12/88**	2/00
**19/11**	2/00
**20/10**	2/00
**20/48**	2/00
	2/00
**16/40**	2/00
**17/25**	2/00
**17/13**	2/00
**18/40**	2/00
**18/89**	2/00
**16/60**	2/00
**15/19**	2/00
	2/00
**19/19**	2/00
**11/63**	2/00
**16/60**	2/00
**17/14**	2/00
**19/77**	2/00

M1
**17/28**
**2/48**

Krusl

Krusl

1
2
3
4

**19/44**	2/00
**17/08**	2/00
**17/25**	2/00
**20/86**	2/00
**19/25**	2/00
**20/42**	2/00
**18/96**	2/00
**16/51**	2/00
	2/00
**17/03**	2/00
**17/68**	3/00
**19/52**	3/00
	3/00
**20/00**	3/00
**17/75**	3/00
**16/34**	3/00
**19/50**	3/00
**16/92**	3/00
**17/39**	3/00
**19/04**	3/00
**14/90**	3/00
	3/00
	3/00
**19/83**	3/00
**20/13**	3/00
**17/56**	3/00
**19/71**	3/00
**18/55**	3/00
**19/98**	3/00
**18/08**	3/00
**16/17**	3/00
**20/23**	3/00
**18/75**	3/00
**19/17**	3/00
**17/50**	3/00
**15/77**	3/00
	3/00
**17/65**	3/00
**16/18**	4/00
**20/68**	4/00
	4/00
**22/60**	4/00
**21/00**	4/00
**17/00**	4/00
	4/00
**18/78**	4/00
**20/00**	4/00

**20/05**	4/00
**15/54**	4/00
	4/00
	4/00
**20/00**	4/00
**20/10**	4/00
**17/19**	4/00
**18/79**	4/00
**19/00**	4/00
**20/06**	4/00
**17/89**	4/00
**17/00**	4/00
**20/64**	4/00
**17/06**	4/00
**19/75**	4/00
**24/00**	4/00
**16/30**	4/00
**14/75**	4/00
**17/65**	4/00

M2	M3	M4
17/74	18/25	18/83
2/26	1/52	2/25

kal-Wallis ANOVA by Ranks; Var1 (Spreadsheet1)
Independent (grouping) variable: Var2
kal-Wallis test: H (3, N= 91) =6,255347 p =,0998

Code	Valid	Sum of	Mean
1	18	615/500	34/19444
2	25	1108/000	44/32000
3	24	1159/000	48/29167
4	24	1303/500	54/31250

Dur
97/00
84/00
110/00
110/00
97/00
97/00
84/00
51/00
84/00
94/33
84/00
80/40
85/38
70/06
59/80
52/42
68/30
68/71
66/18
70/98
56/87
80/27
82/92
57/67
69/17
72/67
60/38

85/84
64/67
72/29
86/22
77/42
66/16
74/18
54/68

65/08
67/25
82/04

65/49
66/27
52/88
66/35
77/40
59/59
72/67
55/98

61/73
56/23
63/55
62/69
76/13
75/50
67/81
51/27
59/31
59/29
59/44
88/78
52/77
115/00
83/78
75/53
75/94

68/94

67/00
53/63
74/57
67/42

79/47
68/93
49/00

76/22
69/92
72/75
76/05
76/76
74/63
76/44
62/80
77/25
63/46
72/35
75/40
73/78
81/87
77/30

Microcycle
1/00
1/00
1/00
1/00
1/00
1/00
1/00
1/00
1/00
1/00
1/00
1/00
1/00
1/00
1/00
1/00
1/00
1/00
1/00
1/00
1/00
1/00
1/00
1/00
1/00
1/00
1/00
1/00
2/00
2/00
2/00
2/00
2/00
2/00
2/00
2/00
2/00
2/00
2/00
2/00
2/00
2/00
2/00
2/00
2/00
2/00

M1	M2	M3	M4
90/21	69/95	67/97	71/50
16/22	9/93	14/05	7/72

Kruskal-Wallis ANOVA by Ranks; Var1 (Spreads
Independent (grouping) variable: Var2
Kruskal-Wallis test: H (3, N= 86) =19,09831 p =,

	Code	Valid	Sum of
1	1	11	791/000
2	2	25	1002/000
3	3	25	828/000
4	4	25	1120/000

2/00
2/00
2/00
2/00
2/00
2/00
2/00
2/00
2/00
2/00
3/00
3/00
3/00
3/00
3/00
3/00
3/00
3/00
3/00
3/00
3/00
3/00
3/00
3/00
3/00
3/00
3/00
3/00
3/00
3/00
3/00
3/00
3/00
3/00
3/00
3/00
3/00
3/00
4/00
4/00
4/00
4/00
4/00
4/00
4/00
4/00
4/00

4/00
4/00
4/00
4/00
4/00
4/00
4/00
4/00
4/00
4/00
4/00
4/00
4/00
4/00
4/00
4/00
4/00
4/00
4/00

heet1)

0003

Mean
71/90909
40/08000
33/12000
44/80000

RPE	Microcycle
**6/48**	1/00
**5/83**	1/00
**5/92**	1/00
**7/88**	1/00
	1/00
**7/06**	1/00
**8/33**	1/00
	1/00
**8/03**	1/00
**6/33**	1/00
**7/19**	1/00
**6/00**	1/00
**6/08**	1/00
**7/08**	1/00
	1/00
**6/44**	1/00
**7/30**	1/00
	1/00
**7/79**	1/00
**7/33**	1/00
**7/30**	1/00
**6/52**	1/00
	1/00
	1/00
	1/00
**6/00**	1/00
	1/00
	1/00
**6/63**	2/00
**6/06**	2/00
**6/80**	2/00
**6/13**	2/00
	2/00
**6/08**	2/00
**5/92**	2/00
**6/03**	2/00
**5/13**	2/00
**6/08**	2/00
**5/77**	2/00
**6/44**	2/00
	2/00
**5/33**	2/00
**6/08**	2/00
**4/71**	2/00
**5/59**	2/00
**4/77**	2/00

M1
**6/89**
**0/78**

Krusl

Krus

1
2
3
4

**5/41**	2/00
**3/42**	2/00
**6/21**	2/00
**5/49**	2/00
**6/54**	2/00
**4/34**	2/00
**5/72**	2/00
**5/81**	2/00
	2/00
**7/22**	2/00
**5/42**	3/00
**5/38**	3/00
	3/00
**6/42**	3/00
**6/22**	3/00
**5/00**	3/00
**6/67**	3/00
**5/71**	3/00
**6/00**	3/00
**5/92**	3/00
**5/70**	3/00
	3/00
	3/00
**4/10**	3/00
**5/33**	3/00
**4/91**	3/00
**4/96**	3/00
**3/88**	3/00
**4/50**	3/00
**5/56**	3/00
**5/39**	3/00
**5/15**	3/00
**6/75**	3/00
**5/09**	3/00
**7/58**	3/00
**6/83**	3/00
	3/00
**7/61**	3/00
**5/08**	4/00
**4/64**	4/00
	4/00
**5/60**	4/00
	4/00
**4/08**	4/00
**8/00**	4/00
**5/18**	4/00
**5/75**	4/00

**4/92**	4/00
**6/08**	4/00
	4/00
	4/00
**4/20**	4/00
**5/56**	4/00
**5/30**	4/00
**5/85**	4/00
**3/43**	4/00
**5/66**	4/00
**4/06**	4/00
**4/44**	4/00
**5/57**	4/00
**5/19**	4/00
**4/45**	4/00
**4/78**	4/00
**6/98**	4/00
**3/96**	4/00
**5/96**	4/00

M2	M3	M4
5/75	5/67	5/20
0/82	0/97	1/01

kal-Wallis ANOVA by Ranks; Var1 (Spreadsheet1)
Independent (grouping) variable: Var2
kal-Wallis test: H (3, N= 92) =29,44080 p =,0000

Code	Valid	Sum of	Mean
1	19	1394/500	73/39474
2	25	1167/000	46/68000
3	24	1001/500	41/72917
4	24	715/000	29/79167

ITL
799/00
756/00
926/00
1022/67
841/00
737/33
756/00
459/00
781/67
773/33
756/00
611/64
516/06
539/58
487/50
458/83
474/25
441/56
445/50
418/63
754/83
625/08
466/67
533/23
429/42
360/10

634/25
444/17
545/00
643/94
518/11
363/57
467/50
375/28

440/42
390/51
447/67

425/83
396/22
408/88
396/83
574/23
412/67
444/35
435/28

266/19
351/36
337/13
340/76
502/06
502/56
345/13
363/67
365/36
436/08
342/51
657/00
442/06

696/50
423/33
420/70

497/56

302/38
248/00
469/46
269/25

429/11
439/06

341/60
399/54
514/66
445/70
330/02
502/25
372/03
348/83
447/18
378/17
353/08
443/29
588/94
294/60
468/64

Microcycle
1/00
1/00
1/00
1/00
1/00
1/00
1/00
1/00
1/00
1/00
1/00
1/00
1/00
1/00
1/00
1/00
1/00
1/00
1/00
1/00
1/00
1/00
1/00
1/00
1/00
1/00
1/00
1/00
2/00
2/00
2/00
2/00
2/00
2/00
2/00
2/00
2/00
2/00
2/00
2/00
2/00
2/00
2/00
2/00
2/00
2/00

M1	M2	M3	M4
782/55	499/80	428/37	405/31
138/15	97/84	100/45	84/04

Kruskal-Wallis ANOVA by Ranks; Var1 (Spreads
Independent (grouping) variable: Var2
Kruskal-Wallis test: H (3, N= 83) =34,26829 p =,

	Code	Valid	Sum of
1	1	11	832/000
2	2	24	1182/000
3	3	24	757/000
4	4	24	715/000

2/00
2/00
2/00
2/00
2/00
2/00
2/00
2/00
2/00
2/00
3/00
3/00
3/00
3/00
3/00
3/00
3/00
3/00
3/00
3/00
3/00
3/00
3/00
3/00
3/00
3/00
3/00
3/00
3/00
3/00
3/00
3/00
3/00
3/00
3/00
3/00
3/00
3/00
4/00
4/00
4/00
4/00
4/00
4/00
4/00
4/00
4/00

4/00
4/00
4/00
4/00
4/00
4/00
4/00
4/00
4/00
4/00
4/00
4/00
4/00
4/00
4/00
4/00
4/00
4/00
4/00

heet1)
0000
Mean
75/63636
49/25000
31/54167
29/79167

TD	Microcycle
**9547/70**	1/00
	1/00
	1/00
	1/00
	1/00
**7154/11**	1/00
**7995/51**	1/00
	1/00
**8657/16**	1/00
	1/00
**8488/00**	1/00
	1/00
	1/00
**8872/27**	1/00
	1/00
**7842/40**	1/00
	1/00
**4379/56**	1/00
**7143/28**	1/00
	1/00
**8194/79**	1/00
	1/00
	1/00
	1/00
	1/00
**8006/00**	1/00
	1/00
	1/00
**8422/97**	2/00
	2/00
	2/00
**5935/84**	2/00
**3951/05**	2/00
**3891/52**	2/00
**5713/46**	2/00
**5860/69**	2/00
**6185/22**	2/00
	2/00
**5273/72**	2/00
**8981/14**	2/00
	2/00
**8370/31**	2/00
**3972/00**	2/00
**6425/43**	2/00
**6809/02**	2/00
**5411/12**	2/00

M1
**7843/71**
**1348/24**

Krusl
Krus
1
2
3
4

**8057/83**	2/00
**5900/46**	2/00
**6514/26**	2/00
**7927/81**	2/00
**7194/86**	2/00
**6543/46**	2/00
**6503/46**	2/00
**4889/08**	2/00
	2/00
**4802/96**	2/00
**5083/56**	3/00
	3/00
	3/00
**5360/75**	3/00
**4471/60**	3/00
**3522/27**	3/00
**5723/86**	3/00
**6131/21**	3/00
**5149/76**	3/00
	3/00
**4252/26**	3/00
	3/00
	3/00
**5241/52**	3/00
**4446/00**	3/00
**5428/28**	3/00
**5053/90**	3/00
**6619/24**	3/00
**5818/48**	3/00
**4570/74**	3/00
**4210/70**	3/00
**4418/85**	3/00
**5208/88**	3/00
**4762/76**	3/00
**9992/03**	3/00
**4254/14**	3/00
	3/00
**7605/35**	3/00
**6285/84**	4/00
	4/00
	4/00
**4648/00**	4/00
	4/00
**4345/31**	4/00
**3722/66**	4/00
**4866/59**	4/00
**5505/18**	4/00

	4/00
**4900/01**	4/00
	4/00
	4/00
**6515/61**	4/00
**6010/22**	4/00
**5765/12**	4/00
**6189/37**	4/00
**6057/70**	4/00
**5820/90**	4/00
**5208/69**	4/00
**4669/42**	4/00
**5770/93**	4/00
**5033/35**	4/00
**6153/37**	4/00
**4739/38**	4/00
**5972/76**	4/00
	4/00
**6419/00**	4/00

M2	M3	M4
6240/77	5333/01	5457/11
1454/12	1378/38	772/95

kal-Wallis ANOVA by Ranks; Var1 (Spreadsheet1)
Independent (grouping) variable: Var2
kal-Wallis test: H (3, N= 77) =21,03442 p =,0001

Code	Valid	Sum of	Mean
1	11	692/000	62/90909
2	23	1013/000	44/04348
3	22	606/000	27/54545
4	21	692/000	32/95238

PL
1065/70
558/90
766/04
988/02
1063/97
829/00
807/24
615/00
736/54
820/09
749/00
892/13
599/61
464/27
378/27
527/53
626/26
671/34
657/14
775/12
748/27
419/00
688/08
840/47
634/29

811/57
563/93
712/44
779/42
864/58
705/21
659/27
508/67

521/29
616/46

576/17
510/03
350/85
544/34
660/05
559/46

524/34

496/40
506/39
566/38
642/65
770/78
585/85
440/37
465/26
459/06
633/79
530/82
997/02
445/69

846/81
700/63

487/00

411/83
387/61
559/64
586/17

585/20

588/31
641/23
646/21
768/00
698/47
594/75
498/14
548/68
583/57
602/18
641/69
553/39
618/14

726/65

Microcycle
1/00
1/00
1/00
1/00
1/00
1/00
1/00
1/00
1/00
1/00
1/00
1/00
1/00
1/00
1/00
1/00
1/00
1/00
1/00
1/00
1/00
1/00
1/00
1/00
1/00
1/00
1/00
1/00
2/00
2/00
2/00
2/00
2/00
2/00
2/00
2/00
2/00
2/00
2/00
2/00
2/00
2/00
2/00
2/00
2/00
2/00

M1	M2	M3	M4
**818/14**	**654/27**	**578/59**	**591/79**
**165/36**	**141/69**	**144/52**	**94/74**

Kruskal-Wallis ANOVA by Ranks; Var1 (Spreads

Independent (grouping) variable: Var2

Kruskal-Wallis test: H (3, N= 77) =17,32760 p =,

	Code	Valid	Sum of
1	1	11	671/0000
2	2	23	995/0000
3	3	22	633/0000
4	4	21	704/0000

2/00
2/00
2/00
2/00
2/00
2/00
2/00
2/00
2/00
2/00
3/00
3/00
3/00
3/00
3/00
3/00
3/00
3/00
3/00
3/00
3/00
3/00
3/00
3/00
3/00
3/00
3/00
3/00
3/00
3/00
3/00
3/00
3/00
3/00
3/00
3/00
3/00
3/00
4/00
4/00
4/00
4/00
4/00
4/00
4/00
4/00
4/00

4/00
4/00
4/00
4/00
4/00
4/00
4/00
4/00
4/00
4/00
4/00
4/00
4/00
4/00
4/00
4/00
4/00
4/00
4/00

heet1)

0006

Mean
61/00000
43/26087
28/77273
33/52381

ACC	Microcycle
**22/00**	1/00
	1/00
	1/00
	1/00
	1/00
**21/00**	1/00
**15/33**	1/00
	1/00
**22/67**	1/00
	1/00
**20/00**	1/00
	1/00
	1/00
**23/33**	1/00
	1/00
**31/00**	1/00
	1/00
**10/00**	1/00
**14/17**	1/00
	1/00
**17/83**	1/00
	1/00
	1/00
	1/00
	1/00
**17/00**	1/00
	1/00
	1/00
**21/93**	2/00
	2/00
	2/00
**18/89**	2/00
**10/00**	2/00
**14/61**	2/00
**16/58**	2/00
**11/92**	2/00
**15/41**	2/00
	2/00
**14/25**	2/00
**25/83**	2/00
	2/00
**28/52**	2/00
**8/00**	2/00
**28/25**	2/00
**29/50**	2/00
**11/96**	2/00

M1
**19/48**
**5/57**

Krusl

Krus

1
2
3
4

**23/17**	2/00
**22/22**	2/00
**16/61**	2/00
**29/94**	2/00
**17/81**	2/00
**20/88**	2/00
**15/42**	2/00
**21/20**	2/00
	2/00
**22/54**	2/00
**18/71**	3/00
	3/00
	3/00
**15/19**	3/00
**16/06**	3/00
**12/60**	3/00
**17/85**	3/00
**11/95**	3/00
**17/21**	3/00
	3/00
**10/19**	3/00
	3/00
	3/00
**18/46**	3/00
**12/00**	3/00
**24/84**	3/00
**18/08**	3/00
**10/18**	3/00
**14/71**	3/00
**13/44**	3/00
**11/13**	3/00
**20/44**	3/00
**14/27**	3/00
**20/60**	3/00
**21/67**	3/00
**18/92**	3/00
	3/00
**28/13**	3/00
**21/17**	4/00
	4/00
	4/00
**17/00**	4/00
	4/00
**17/75**	4/00
**11/88**	4/00
**14/64**	4/00
**16/91**	4/00

	4/00
**13/33**	4/00
	4/00
	4/00
**20/69**	4/00
**18/75**	4/00
**20/16**	4/00
**23/05**	4/00
**11/85**	4/00
**18/37**	4/00
**18/70**	4/00
**15/73**	4/00
**22/15**	4/00
**15/25**	4/00
**22/55**	4/00
**19/43**	4/00
**26/39**	4/00
	4/00
**42/58**	4/00

M2	M3	M4
19/37	16/67	19/44
6/36	4/71	6/49

kal-Wallis ANOVA by Ranks; Var1 (Spreadsheet1)
Independent (grouping) variable: Var2
kal-Wallis test: H (3, N= 77) =3,603580 p =,3076

Code	Valid	Sum of	Mean
1	11	480/0000	43/63636
2	23	969/5000	42/15217
3	22	692/0000	31/45455
4	21	861/5000	41/02381

DCC
30/67
20/00
11/00
22/00
19/00
32/00
39/00
9/00
21/17
26/00
31/00
33/38
17/06
10/00
13/71
21/87
14/08
20/55
16/42
39/67
40/19
17/00
39/35
27/67
20/83

28/92
24/22
27/40
30/82
22/27
25/86
21/50
28/56

19/58
25/00

15/19
15/56
10/56
22/26
16/69
19/97

10/13

20/38
14/00
29/81
17/99
17/50
17/38
15/88
16/70
21/51
18/70
18/51
37/78
19/24

33/96
29/90

16/33

13/38
13/88
13/62
18/19

8/15

27/22
22/58
28/43
22/40
16/83
18/85
16/94
21/57
26/20
17/28
23/95
17/15
26/29

28/07

Microcycle
1/00
1/00
1/00
1/00
1/00
1/00
1/00
1/00
1/00
1/00
1/00
1/00
1/00
1/00
1/00
1/00
1/00
1/00
1/00
1/00
1/00
1/00
1/00
1/00
1/00
1/00
1/00
1/00
2/00
2/00
2/00
2/00
2/00
2/00
2/00
2/00
2/00
2/00
2/00
2/00
2/00
2/00
2/00
2/00
2/00
2/00

M1	M2	M3	M4
**23/71**	**24/39**	**19/76**	**20/34**
**9/12**	**8/41**	**6/78**	**5/95**

Kruskal-Wallis ANOVA by Ranks; Var1 (Spreads
Independent (grouping) variable: Var2
Kruskal-Wallis test: H (3, N= 77) =5,760254 p =,

	Code	Valid	Sum of
1	1	11	500/000
2	2	23	1054/000
3	3	22	702/000
4	4	21	747/000

2/00
2/00
2/00
2/00
2/00
2/00
2/00
2/00
2/00
2/00
3/00
3/00
3/00
3/00
3/00
3/00
3/00
3/00
3/00
3/00
3/00
3/00
3/00
3/00
3/00
3/00
3/00
3/00
3/00
3/00
3/00
3/00
3/00
3/00
3/00
3/00
3/00
3/00
4/00
4/00
4/00
4/00
4/00
4/00
4/00
4/00
4/00

4/00
4/00
4/00
4/00
4/00
4/00
4/00
4/00
4/00
4/00
4/00
4/00
4/00
4/00
4/00
4/00
4/00
4/00
4/00

heet1)

1239

Mean
45/45455
45/82609
31/90909
35/57143

RHI	Microcycle
**243/39**	1/00
	1/00
	1/00
	1/00
	1/00
	1/00
**308/32**	1/00
**130/01**	1/00
	1/00
**165/79**	1/00
	1/00
**244/09**	1/00
	1/00
	1/00
**447/29**	1/00
	1/00
**599/32**	1/00
	1/00
**48/28**	1/00
**292/72**	1/00
	1/00
**298/39**	1/00
	1/00
	1/00
	1/00
	1/00
**459/00**	1/00
	1/00
**317/75**	2/00
	2/00
	2/00
	2/00
**278/95**	2/00
**293/35**	2/00
**145/67**	2/00
**216/07**	2/00
**167/29**	2/00
**300/54**	2/00
	2/00
**253/08**	2/00
**497/33**	2/00
	2/00
**554/67**	2/00
**251/00**	2/00
**399/00**	2/00
**277/44**	2/00

M1
**294/24**
**159/04**

Krusl

Krus

1
2
3
4

**182/86**	2/00
**317/82**	2/00
**216/69**	2/00
**174/70**	2/00
**300/99**	2/00
**296/57**	2/00
**257/43**	2/00
**236/10**	2/00
**286/86**	2/00
	2/00
**116/99**	3/00
	3/00
	3/00
	3/00
**212/61**	3/00
**89/69**	3/00
**97/00**	3/00
**279/98**	3/00
**175/64**	3/00
**274/67**	3/00
	3/00
**143/45**	3/00
	3/00
	3/00
**244/53**	3/00
**171/68**	3/00
**327/32**	3/00
**164/18**	3/00
**184/23**	3/00
**183/61**	3/00
**124/21**	3/00
**172/62**	3/00
**149/44**	3/00
**191/59**	3/00
**143/36**	3/00
**464/72**	3/00
**205/84**	3/00
	3/00
**237/42**	4/00
	4/00
	4/00
**209/00**	4/00
**235/00**	4/00
	4/00
**107/59**	4/00
**127/63**	4/00
**141/91**	4/00

**232/25**	4/00
	4/00
**146/84**	4/00
	4/00
	4/00
**346/34**	4/00
**340/28**	4/00
**318/95**	4/00
**295/47**	4/00
**153/39**	4/00
**202/79**	4/00
**169/49**	4/00
**156/13**	4/00
**207/81**	4/00
**177/93**	4/00
**223/73**	4/00
**115/75**	4/00
**269/90**	4/00
	4/00

M2	M3	M4
282/83	196/07	210/27
98/62	86/17	72/11

kal-Wallis ANOVA by Ranks; Var1 (Spreadsheet1)
Independent (grouping) variable: Var2
kal-Wallis test: H (3, N= 75) =12,86465 p =,0049

Code	Valid	Sum of	Mean
1	11	508/000	46/18182
2	22	1074/000	48/81818
3	21	584/000	27/80952
4	21	684/000	32/57143

SP
45/91
74/68
7/24
10/35
66/20
155/22
320/28
0/00
89/23
53/37
156/00
56/85
81/12
63/01
37/73
39/52
35/60
60/18
28/63
99/12
178/96
75/00
127/99
35/58

7/14
81/22
84/42
33/68
57/74
44/15
17/74
35/18
77/21

20/80

61/64
19/16
17/98
77/65
29/74
75/70

26/35

65/71
36/09
110/76
15/00
21/00
40/44
27/90
50/58
21/38
26/08
15/02
48/89
51/20

51/80

35/50
73/67

21/86
47/75
24/28

49/89

23/05

120/67
72/31
107/16
41/02
41/62
47/53
50/51
36/93
56/06
32/90
28/22
43/96
104/28

Microcycle	M1	M2	M3	M4
1/00	88/95	61/72	40/91	52/90
1/00	93/28	38/74	25/51	27/98
1/00				
1/00				
1/00				
1/00				
1/00				
1/00				
1/00				
1/00				
1/00				
1/00				
1/00				
1/00				
1/00				
1/00				
1/00				
1/00				
1/00				
1/00				
1/00				
1/00				
1/00				
1/00				
1/00				
1/00				
1/00				
1/00				
2/00				
2/00				
2/00				
2/00				
2/00				
2/00				
2/00				
2/00				
2/00				
2/00				
2/00				
2/00				
2/00				
2/00				
2/00				
2/00				
2/00				
2/00				

Kruskal-Wallis ANOVA by Ranks; Var1 (Spreads

Independent (grouping) variable: Var2

Kruskal-Wallis test: H (3, N= 75) =5,445992 p =,

	Code	Valid	Sum of
1	1	11	487/0000
2	2	22	943/0000
3	3	21	613/0000
4	4	21	807/0000

2/00
2/00
2/00
2/00
2/00
2/00
2/00
2/00
2/00
2/00
3/00
3/00
3/00
3/00
3/00
3/00
3/00
3/00
3/00
3/00
3/00
3/00
3/00
3/00
3/00
3/00
3/00
3/00
3/00
3/00
3/00
3/00
3/00
3/00
3/00
3/00
3/00
3/00
4/00
4/00
4/00
4/00
4/00
4/00
4/00
4/00
4/00

4/00
4/00
4/00
4/00
4/00
4/00
4/00
4/00
4/00
4/00
4/00
4/00
4/00
4/00
4/00
4/00
4/00
4/00
4/00

heet1)	
1419	
Mean	
44/27273	
42/86364	
29/19048	
38/42857	

MAX S	Microcycle
**32/75**	1/00
	1/00
	1/00
	1/00
	1/00
	1/00
**30/08**	1/00
**34/60**	1/00
	1/00
**26/35**	1/00
	1/00
**26/90**	1/00
	1/00
	1/00
**29/58**	1/00
	1/00
**32/60**	1/00
	1/00
**21/51**	1/00
**27/02**	1/00
	1/00
**29/02**	1/00
	1/00
	1/00
	1/00
	1/00
**30/90**	1/00
	1/00
**27/44**	2/00
	2/00
	2/00
	2/00
**28/29**	2/00
**26/87**	2/00
**27/14**	2/00
**26/98**	2/00
**26/18**	2/00
**28/19**	2/00
	2/00
**26/78**	2/00
**29/61**	2/00
	2/00
**30/37**	2/00
**29/60**	2/00
**30/05**	2/00
**26/72**	2/00

M1
**29/21**
**3/67**

Krusl
Krus
1
2
3
4

**24/94**	2/00
**28/05**	2/00
**28/71**	2/00
**27/71**	2/00
**27/42**	2/00
**27/88**	2/00
**26/11**	2/00
**25/93**	2/00
**29/03**	2/00
	2/00
**26/69**	3/00
	3/00
	3/00
	3/00
**27/24**	3/00
**27/06**	3/00
**26/48**	3/00
**28/37**	3/00
**26/24**	3/00
**28/06**	3/00
	3/00
**26/32**	3/00
	3/00
	3/00
**27/44**	3/00
**26/51**	3/00
**29/15**	3/00
**26/00**	3/00
**25/56**	3/00
**26/16**	3/00
**26/47**	3/00
**28/94**	3/00
**26/48**	3/00
**27/36**	3/00
**25/15**	3/00
**27/12**	3/00
**28/64**	3/00
	3/00
**28/53**	4/00
	4/00
	4/00
**26/90**	4/00
**31/60**	4/00
	4/00
**26/70**	4/00
**24/12**	4/00
**25/21**	4/00

**28/31**	4/00
	4/00
**25/80**	4/00
	4/00
	4/00
**29/34**	4/00
**28/02**	4/00
**29/72**	4/00
**27/12**	4/00
**26/74**	4/00
**27/31**	4/00
**27/55**	4/00
**26/97**	4/00
**27/69**	4/00
**27/41**	4/00
**26/64**	4/00
**26/06**	4/00
**29/49**	4/00
	4/00

M2	M3	M4
27/73	27/02	27/49
1/42	1/09	1/67

kal-Wallis ANOVA by Ranks; Var1 (Spreadsheet1)
Independent (grouping) variable: Var2
kal-Wallis test: H (3, N= 75) =7,584835 p =,0554

Code	Valid	Sum of	Mean
1	11	563/0000	51/18182
2	22	894/0000	40/63636
3	21	620/0000	29/52381
4	21	773/0000	36/80952
